# Enhancing the sensitivity of rapid antigen detection test (RADT) of different SARS-CoV-2 variants and lineages using fluorescence-labeled antibodies and a fluorescent meter

**DOI:** 10.1016/j.heliyon.2023.e17179

**Published:** 2023-06-10

**Authors:** Gheyath K. Nasrallah, Fatma Ali, Salma Younes, Heba A. Al Khatib, Asmaa A. Al-Thani, Hadi M. Yassine

**Affiliations:** a Biomedical Research Center, Qatar University, Doha, 2713, Qatar; b Biomedical Sciences Department, College of Health Sciences, Qatar University, Doha, 2713, Qatar

**Keywords:** RADT, SARS-CoV-2, COVID-19, Lateral flow, Immunofluorescence

## Abstract

RT-qPCR is considered the gold standard for diagnosis of COVID-19; however, it is laborious, time-consuming, and expensive. RADTs have evolved recently as relatively inexpensive methods to address these shortcomings, but their performance for detecting different SARS-COV-2 variants remains limited. RADT test performance could be enhanced using different antibody labeling and signal detection techniques. Here, we aimed to evaluate the performance of two antigen RADTs for detecting different SARS-CoV-2 variants: (i) the conventional colorimetric RADT (Ab-conjugated with gold beads); and (ii) the new Finecare™ RADT (Ab-coated fluorescent beads). Finecare™ is a meter used for the detection of a fluorescent signal. 187 frozen nasopharyngeal swabs collected in Universal transport (UTM) that are RT-qPCR positive for different SARS-CoV-2 variants were selected, including Alpha (n = 60), Delta (n = 59), and Omicron variants (n = 108). Sixty flu and 60 RSV-positive samples were included as negative controls (total sample number = 347). The conventional RADT showed sensitivity, specificity, positive predictive value (PPV), and negative predictive value (NPV) of 62.4% (95%CI: 54–70), 100% (95%CI: 97–100), 100% (95%CI: 100-100), and 58% (95%CI: 49–67), respectively. These measurements were enhanced using the Finecare™ RADT: sensitivity, specificity, PPV, and NPV were 92.6% (95%CI: 89.08–92.3), 96% (95%CI: 96–99.61), 98% (95%CI: 89–92.3), and 85% (95%CI: 96–99.6) respectively. The sensitivity of both RADTs could be greatly underestimated because nasopharyngeal swab samples collected UTM and stored at −80 °C were used. Despite that, our results indicate that the Finecare™ RADT is appropriate for clinical laboratory and community-based surveillance due to its high sensitivity and specificity.

## 1 Introduction

In 2021, the WHO has designated a new SARS-CoV-2 variant of concern and interest (VOC/I), including Delta (B.1.617.2), Lambda (C37), Mu (B.1.621), and Omicron (B.1.1.529) variant [1]. The emergence of novel SARS-CoV-2 variants has prompted concerns about increased infectivity, outbreaks among vaccinated people, and the feasibility of current test strategies [2]. Currently, quantitative real-time reverse transcription polymerase chain reaction (RT-qPCR) is considered the gold standard for SARS-CoV-2 diagnosis [3]. Despite its superior clinical performance, RT-qPCR is challenging to implement due to its expensive reagents, longer time to result, and requirements for sophisticated instrumentation [[2], [3], [4]].

Although not as sensitive as RT-qPCR, rapid antigen detection tests (RADTs) based on lateral flow immunoassay (LFIA) technology provide a fast, inexpensive, portable, and effective method of testing in the laboratory and non-laboratory settings [4]. RADTs have been successfully implemented to control HIV and malaria [5] and are quickly becoming an essential tool in SARS-CoV-2 testing [6]. The laboratory evaluations [[7], [8], [9]], and a Cochrane meta-analysis [10], have revealed a highly variable performance of RADTs, resulting in an ongoing debate over the utility of these tests for the detection of acute SARS-CoV-2-infection Several RADTs have already been approved for clinical use. According to WHO, it is recommended to have 80% sensitivity (true positive) and ≥97% specificity (true negative) for RADTs [11]. However, the emergence of the new variant has dramatically decreased the sensitivity of the RADT assays [12,13]. Therefore, to increase the sensitivity of the conventional RADT assay for the detection of SARS-CoV-2 “N” antigen, Guangzhou Wondfo Biotech Co., Ltd. Has developed a new Finecare™ RADT. The new Finecare™ RADT is an immunofluorescent-based assay, where the *anti*-N detecting antibodies are labeled or conjugated with fluorescent nanobeads. Finecare™ is a very sensitive meter used for the detection of the fluorescent signal and results interpretation of different RADT, qualitatively and quantitatively, including hemoglobin A1c (HBA1c), thyroid stimulating hormone (TSH), Beta-human chorionic gonadotropin (B-HCG), etc. However, the Wondfo conventional RADT is a colorimetric and qualitative assay where the *anti*-N detecting antibodies are labeled or conjugated with colloidal gold nanobeads. The test result of the conventional RADT is read out visually. The current study aimed to evaluate the performance of the conventional and the Fincecare RADT in detecting different SARS-CoV-2 variants.

## 2 Methods

### 2.1 Sample collection

This study was conducted in full accordance with the research regulation at Hamad Medical Corporation (HMC) and Qatar University (QU). HMC-Institutional Review Board (HMC-IRB approval MRC-01-20-145) and QU-IRB (QU-IRB 1289-EA/20) reviewed and approved the study. The ethics committee waived the requirement of written informed consent for participation. Nasopharyngeal swabs suspended in universal transport media (UTM) from individuals confirmed with SARS-CoV-2 infection by RT-qPCR were received from the HMC virology lab between May 29, 2020, and January 22, 2022. The cycle threshold (CT) results of the RT-qPCR of each sample were also provided. The CT-values were below 30 ranging from (11.58–29.46), as it was reported that higher CT-values (≥30) are not infectious [14,15]. Viral transport media were received at Qatar University Biomedical Research Center (QU-BRC) and subjected to sequencing, and then stored at −80 °C. 187 UTM that are positive with different variants were selected for RADT, comprising 60 Alpha, 59 multiple Delta, and 108 multiple Omicron variants (see variant details in Table 3). A total of 60 flu and 60 RSV-positive samples were included as negative controls, making the overall sample total number 347.

**Table 1:** RADT results.

		Conventional RADT (n = 347)		Finecare™ RADT (n = 326)	
		Positive	Negative	Positive	Negative
RSV (Conventional RADT n = 60, Finecare™ RADT n = 39)		0 (0%)	60 (100%)	3 (8%)	36 (92%)
Flu (Conventional RADT n = 60, Finecare™ RADT n = 60)		0 (0%)	60 (100%)	1 (2%)	59 (98%)
SARS-CoV-2	aOmicron (n = 68)	47 (69%)	21 (30.8%)	66 (97.1%)	2 (2.9%)
	Omicron BA.4 (n = 20)	14 (70%)	6 (n = 30)	17 (85%)	3 (15%)
	Omicron BA.5 (n = 20)	14 (70%)	6 (30%)	19 (95%)	1 (5%)
	^+^Delta (n = 59)	30 (50.8%)	29 (49.2%)	59 (100%)	0 (0%)
	Alpha (n = 60)	37 (62%)	23 (39%)	49 (83%)	11 (18.6%)
Total		142 (41%)	205 (59%)	214 (66%)	112 (34%)
Sensitivity (95% CI)		62.4% (54–70)		92.6% (89.08–92.3)	
Specificity (95% CI)		100% (100-100)		96% (96–99.61)	
PPV (95% CI)		100% (97–100)		98% (89–92.3)	
NPV (95% CI)		58% (49–67)		85% (96–99.6)	

a B.1.1.529 (n = 9), BA.1 (n = 16), BA.2 (n = 43, 39.8%); total 68. + See Table 3 for detailed lineage classification.

**Table 2:** Sensitivity of the conventional RADT and Finecare™ RADT by reverse transcription polymerase chain reaction (RT-PCR) cycle threshold (Ct) intervals.

RT-PCR Ct value	n (227)	Conventional RADT				n (227)	Finecare™ RADT			
		Positive	Negative	Sensitivity (95% CI)	False negative ration		Positive	Negative	Sensitivity	False negative ration
≤20	121 (53%)	91 (75%)	30 (25%)	75% (95% CI: 66–82)	25%	121 (53%)	118 (98%)	3 (2%)	98% (95% CI:92–99)	2.5%
20-≤25	89 (39%)	51 (57%)	38 (43%)	57% (95% CI: 46–68)	43%	89 (39%)	81 (91%)	8 (9%)	91% (95% CI:83–96)	9%
>25	17 (7%)	1 (6%)	16 (94%)	6% (95% CI: 0.3–30)	94%	17 (7%)	10 (59%)	7 (41%)	58.8% (95% CI:33–81)	4.1%

**Table 3:** Conventional RADT and Finecare™ RADT detailed test results of SARS-CoV-2 lineages.

Lineage/RADT test	Conventional RADT		Finecare™ RADT	
	Positive	Negative	Positive	Negative
Omicron (n = 108)	75 (69.4%)	33 (30.6%)	102 (94%)	6 (6%)
B.1.1.529 (n = 9, 8.3%)	5 (4.6%)	4 (3.7%)	9 (8.3%)	0 (0%)
BA.1 (n = 16, 14.8%)	8 (7.4%)	8 (7.4%)	14 (13%)	2 (1.9%)
BA.2 (n = 43, 39.8%)	34 (31.5%)	9 (8.3%)	43 (39.8%)	0 (0%)
BA.4 (n = 12, 11.1%)	8 (7.4%)	4 (3.7%)	10 (9.3%)	2 (1.9%)
BA.4.1 (n = 6, 5.6%)	5 (4.6%)	1 (0.9%)	5 (4.6%)	1 (0.9%)
BA.4.5 (n = 2, 1.9%)	1 (0.9%)	1 (0.9%)	2 (1.9%)	0 (0%)
BA.5.1 (n = 3, 2.8%)	2 (1.9%)	1 (0.9%)	3 (2.8%)	0 (0%)
BA.5.2 (n = 14, 13%)	9 (8.3%)	5 (4.6%)	13 (12%)	1 (0.9%)
BA.5.2.1 (n = 2, 1.9%)	2 (1.9%)	0 (0%)	2 (1.9%)	0 (0%)
BA.5.3.1 (n = 1, 0.9%)	1 (0.9%)	0 (0%)	1 (0.9%)	0 (0%)
Delta (n = 59)	30 (50.8%)	29 (49.2%)	59 (100%)	0 (0%)
AY.102 (n = 1, 1.7%)	1 (1.7%)	0 (0%)	1 (1.7%)	0 (0%)
AY.106 (n = 1, 1.7%)	0 (0%)	1 (1.7%)	1 (1.7%)	0 (0%)
AY.114 (n = 1, 1.7%)	0 (0%)	1 (1.7%)	1 (1.7%)	0 (0%)
AY.116 (n = 1, 1.7%)	1 (1.7%)	0 (0%)	1 (1.7%)	0 (0%)
AY.120 (n = 1, 1.7%)	0 (0%)	1 (1.7%)	1 (1.7%)	0 (0%)
AY.122.1 (n = 1, 1.7%)	1 (1.7%)	0 (0%)	1 (1.7%)	0 (0%)
AY.26 (n = 1, 1.7%)	1 (1.7%)	0 (0%)	1 (1.7%)	0 (0%)
AY.32 (n = 1, 1.7%)	1 (1.7%)	0 (0%)	1 (1.7%)	0 (0%)
AY.33 (n = 2, 3.4%)	2 (3.4%)	0 (0%)	2 (3.4%)	0 (0%)
AY.34 (n = 1, 1.7%)	0 (0%)	1 (1.7%)	1 (1.7%)	0 (0%)
AY.4 (n = 13, 22%)	5 (8.5%)	8 (13.5%)	13 (22%)	0 (0%)
AY.43 (n = 2, 3.3%)	1 (1.7%)	1 (1.7%)	2 (3.4%)	0 (0%)
AY.47 (n = 1, 1.7%)	1 (1.7%)	0 (0%)	1 (1.7%)	0 (0%)
AY.5 (n = 1, 1.7%)	1 (1.7%)	0 (0%)	1 (1.7%)	0 (0%)
AY.5.2 (n = 1, 1.7%)	0 (0%)	1 (1.7%)	1 (1.7%)	0 (0%)
AY.9 (n = 1, 1.7%)	1 (1.7%)	0 (0%)	1 (1.7%)	0 (0%)
B.1.617.2 (n = 29, 49.2%)	14 (23.7%)	15 (25.4%)	29 (49.2%)	0 (0%)
Alpha (n = 60)	37 (62%)	23 (38%)	49 (82%)	11 (18%)

### 2.2 Real-time reverse-transcription polymerase chain reaction testing

All PCR testing was conducted at the Hamad Medical Corporation (HMC) Central Laboratory, Sidra Medicine Laboratory, or National reference laboratory (NRL) following standardized protocols. Nasopharyngeal and/or oropharyngeal swabs were collected from patients and placed in UTM. The RNA was extracted using KingFisher Flex (Thermo Fisher Scientific, USA), MGISP-960 (MGI, China), or ExiPrep 96 Lite (Bioneer, South Korea). The real-time reverse-transcription PCR (RT-qPCR) using TaqPath COVID-19 Combo Kits (Thermo Fisher Scientific, USA) on an ABI 7500 FAST (Thermo Fisher Scientific, USA) targeting the viral S, N, and ORF1ab gene regions, tested directly on the Cepheid GeneXpert system using the Xpert Xpress SARS-CoV-2 (Cepheid, USA) targeting the viral N and E gene regions, or loaded directly into a Rochecobas 6800 system and assayed with the cobas SARS-CoV-2 Test (Roche, Switzerland) targeting the ORF1ab and E-gene regions. Results were reported by use of pre-determined gene cut-offs of the respective kit [16]. Both RSV and flu samples were tested by multiplex real-time PCR for detection of pathogen genes by TaqMan technology. Specimens were tested using full panel of respiratory pathogens (Respiratory Pathogens 21 kit, Fast-Track Diagnostics, Luxemburg). This kit enables the identification of 20 viruses and one bacteria that cause upper respiratory tract infections including flu and RSV [17].

### 2.3 Sequencing: determination of SARS-cov-2 variants

The RNA was extracted from UTM using one of the previously described methods. The sequencing was performed at QU-BRC using the MGI G50 test, CleanPlex for MGI SARS-CoV-2. Research and Surveillance Panel (Paragon Genomics, USA), and DNBSEQ-G50RS High-throughput Sequencing Kit (FCL PE100/FCS PE150) [18], were used according to the manufacturer's protocols. Data analysis was conducted using a script, and FASTA fields were submitted to Bioinformatic Pangolin to identify the SARS-CoV-2 lineages [19,20]. All SARS-CoV-2 samples were sequenced to determine SARS-CoV-2 VOC/Is, except for Alpha samples, there were not sequenced. Alpha samples were selected based on the collection date when the Alpha variant was the most prevalent between May and June 2020 [21,22].

### 2.4 Conventional RADT

The conventional RADT (Cat No. W634; Wondfo, China) was performed according to the manufacturer's instructions [23]. In brief, the provided collection swab in the kit was embedded in the viral transport media then the swab was mixed with the provided extraction buffer tube in the kit. Three to four drops (80 μl) were applied through a nozzle cap onto the test strip. The test strip (containing the gold beads labeled Ab) results were read out visually after 15 min. As recommended by the manufacturer's reference guide [23], faint lines were considered positive if the control line was also present.

### 2.5 Finecare™ RADT

Fincare™ RADT (Cat No: W286; Wondfo, China) is an affordable, convenient fluorescent based RADT. In the current study, Fincare™ was performed according to the manufacturer's instructions, as described in section 2.3. The test strip (containing the fluorescent-labeled Ab) results were read after 15 min using the Finecare™ FIA Meter Plus (Wondfo, China). Finecare™ Meter Plus automatically calculates test results and presents them using the cut-off index (COI) unit are given as relative flurescence unites (RFU) [24]. A COI greater than 1.0, was interpreted as positive; A COI less than 1.0, was interpreted as negative. The more SARS-COV-2 “N” antigen in the sample, the higher the signal value scanned by the Finecare™, and the greater the sample reading [25].

### 2.6 Statistical analysis

Data were analyzed using GraphPad Prism 9.3.1 (San Diego, CA, USA). An unpaired T-test was used to compare negative and positive results, and *P*-values ≤0.05 were considered statistically significant. One-way ANOVA was performed to compare the VOC/I RADT results, and *P*-values ≤0.05 were considered statistically significant. A correlation test between cycle threshold (CT) and Finecare™ RADT was performed in all graphs; the significance was × p ≤ 0.05, **p ≤ 0.01, or ***p ≤ 0.001. The sensitivity, specificity, positive predictive value (PPV), negative predictive value (NPV), and Cohen's Kappa (***κ***) of the RADTs were calculated using the RT-PCR as a reference method. Ranging between 0 and 1, a kappa value ≤ 0.40 denotes poor agreement, a value between 0.40 and 0.75 denotes fair/good agreement, and a value ≥ 0.75 denotes excellent agreement [23]. RADTs were subjected to ROC analysis to determine the area under the curve (AUC) for the different SARS-CoV-2 variants [26,27]. Statistically, the larger the AUC, the more precise a diagnostic tool's performance. The following describes the relationship between AUC and diagnostic accuracy: AUC 0.5 suggests no discrimination (ability to diagnose patients with and without the disease or condition based on the test), 0.5–0.6 indicates poor discrimination, 0.6–0.7 indicates sufficient discrimination, 0.7–0.8 is regarded as good, 0.8–0.9 is considered excellent, and >0.9 is outstanding.

## 3 Results

### 3.1 Demographic and clinical information

Among the 347 samples, a total of 187 SARS-CoV-2 variant samples were included in the study, comprising 68 Omicron (median age: 35, IQR: 21–53), 59 Delta (median age: 33, IQR: 18–45), and 60 Alpha (median age: 36, IQR: 29–45) SARS-CoV-2 samples (Table S1). The negative controls included 60 flu (median age: 28, IQR: 9–38) and 60 RSV (median age: 5, IQR: 4–5) positive samples. Omicron lineages included BA.4 (n = 20) and BA.5 (n = 20), as they were the most prevalent Omicron lineages worldwide (Table S2).

### 3.2 Rapid antigen detection tests (RADTs) enhanced by the Finecare™ RADT

Finecare™ RADT yielded more positive results (n = 214, 66%) in comparison to conventional RADT (n = 142, 41%) (Table 1). Moreover, the Finecare™ RADT showed higher sensitivity (92.6%, 95%CI: 89.08–92.3) compared to conventional RADT (62.4%, 95%CI: 54–70). Nevertheless, the conventional RADT showed higher specificity (100%, 95%CI: 100-100) compared to Finecare™ RADT (96%, 95%CI: 96–99.61). Moreover, conventional RADT showed higher PPV (100%, 95%CI: 100-100), but lower NPV (58%, 95%CI: 49–67) when compared to Finecare™ RADT (PPV = 98%, 95%CI: 89–92.3; NPV = 85%, 95%CI: 96–99.6).

The RADTs were then stratified by Ct values, and the sensitivity was investigated in relation to viral load. The sensitivity of both RADTs decreased when the Ct values increased. The conventional RADT showed a sensitivity of 75% for RT-PCR Ct values < 20, moderate sensitivity of 57% for Ct values between 20 and 25, and sensitivity decreased dramatically at Ct values > 25 (6%) (Table 2). Similarly, the Finecare™ RADT showed the highest sensitivity for RT-PCR Ct values < 20 (98%), moderate sensitivity for Ct values between 20 and 25 (91%), and sensitivity decreased dramatically at CT values > 25 (58.8%) (Table 2).

### 3.3 Detection of SARS-cov-2 variants and lineages enhanced by the Finecare™ RADT

**Fig. 1:**
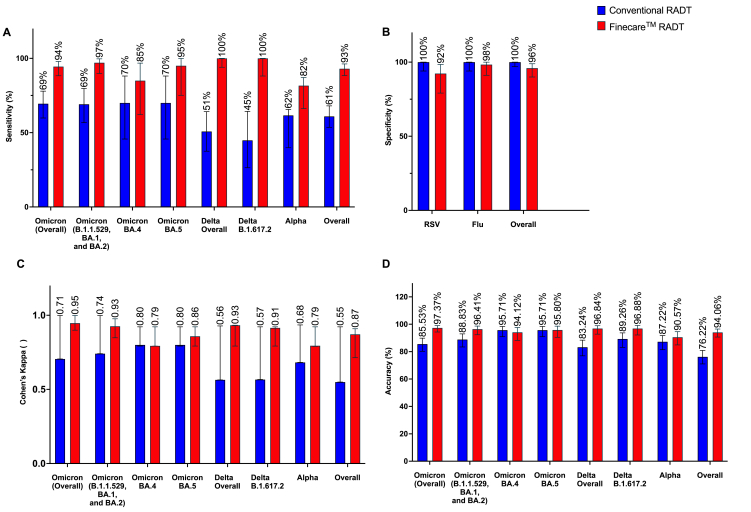
Analytical performance of conventional and Finecare™ RADT. A) Sensitivity % of the two RADTs for the detection of SARS-CoV-2 variants. B) Specificity % among negative controls (RSV and Flu), C) Cohen's kappa (***κ***) score of the two RADTs for the detection of SARS-CoV-2 variants; ***κ*** < 0: no agreement, ***κ*** between 0.00 and 0.20: slight agreement, ***κ*** between 0.21 and 0.40: fair agreement, ***κ*** between 0.41 and 0.60: moderate agreement, ***κ*** between 0.61 and 0.80: substantial agreement, ***κ*** between 0.81 and 1.00: almost perfect agreement. D) Accuracy % of the two RADTs for the detection of SARS-CoV-2 variants. Horizontal black bars indicate the confidence intervals (CIs).

The performance of the conventional RADT and Finecare™ RADT in the detection of SARS-CoV-2 variants was evaluated (Fig. 1). Overall, Finecare™ RADT showed higher sensitivity among all SARS-CoV-2 in comparison to the conventional RADT (Fig. 1A); nevertheless, conventional RADT showed slightly higher specificity (Fig. 1B). Furthermore, the Cohen's kappa (***κ***) score reliability analysis was performed for Finecare™ RADT and conventional RADT using RT-PCR reference method (Fig. 1C). The Cohen's kappa (***κ***) score showed almost perfect agreement between Finecare™ RADT and RT-PCR results with ***κ*** = 0.87 among all SARS-CoV-2 variants. Compare with conventional RADT, which showed fair agreement with ***κ*** = 0.55. The Finecare™ RADT showed higher accuracy among all SARS-CoV-2 in comparison to the conventional RADT (Fig. 1D).

A detailed assessment of the performance of the conventional RADT and the Finecare™ RADT across SARS-CoV-2 lineages was performed (Table 3). Overall, Finecare™ RADT detection of SARS-CoV-2 lineages was higher (92.5%, n = 210 positives) in comparison to the conventional RADT (63%, n = 142 positives) (Table 3). The conventional RADT detected all Delta SARS-CoV-2 lineages, excluding the AY.106, AY.114, AY.120, AY.34, and AY.5.2. With the exception of the AY.34 lineage, the Finecare™ RADT detected all Delta SARS-CoV-2 lineages.

**Fig. 2:**
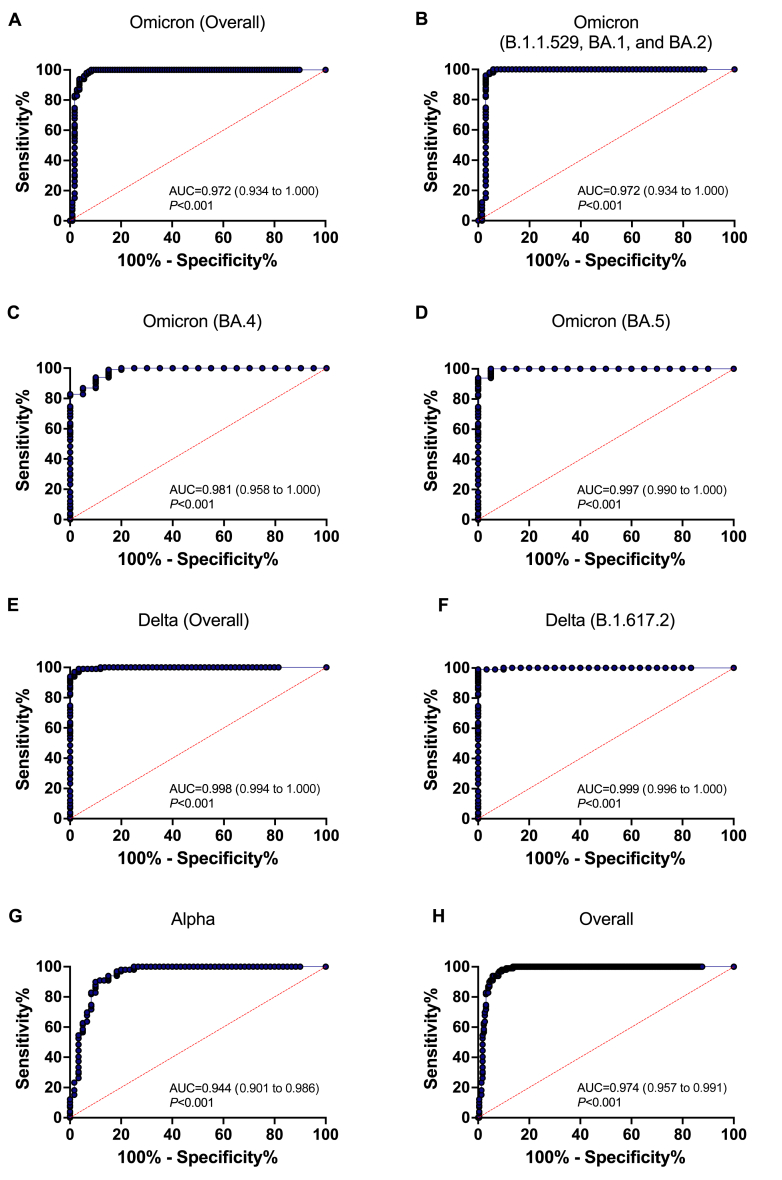
Empirical receiving operating characteristic (ROC) curve analysis for estimating optimal thresholds for Finecare™ RADT against SARS-CoV-2. (A) Finecare™ RADT ROC curve for Omicron-infected patients (n = 108). (B) Finecare™ RADT ROC curve for B.1.1.529, BA.1, and BA.2 Omicron-infected patients (n = 68). (C) Finecare™ RADT ROC curve for BA.4 Omicron-infected patients (n = 20). (D) Finecare™ RADT ROC curve for BA.5 Omicron-infected patients (n = 20). (E) Finecare™ RADT ROC curve for BA.4 Delta-infected patients (n = 59). (F) Finecare™ RADT ROC curve for B.1.617.2 Delta -infected patients (n = 30). (G) Finecare™ RADT ROC curve for Alpha-infected patients (n = 60). (H) Finecare™ RADT ROC curve (overall; n = 227). The sensitivity and specificity values correspond to the plotted points which were used to estimate the area under the curve (AUC) and p-value for each curve.

The empirical receiving operating characteristic (ROC) curve analysis was performed to estimate optimal thresholds for FinecareTM RADT against SARS-CoV-2 (Fig. 2). The ROC AUCs for the assays ranged between 0.934 and 1.000 (AUC = 0.972, p < 0.001), denoting excellent performance for Finecare™ in detecting Omicron variant and Omicron (B.1.1.529, BA.1, and BA.2) (Fig. 2A and B). Similarly, the ROC AUCs for the assays ranged between 0.958 and 1.000 (AUC = 0.981, p < 0.001), denoting excellent performance for Finecare™ in detecting Omicron (BA.4) variant (Fig. 2C). Likewise, the ROC AUCs for the assays ranged between 0.990 and 1.000 (AUC = 0.997, p < 0.001), denoting excellent performance for Finecare™ in detecting Omicron (BA.5) variant (Fig. 2D). The ROC AUCs for the assays ranged between 0.994 and 1.000 (AUC = 0.998, p < 0.001), denoting excellent performance for Finecare™ in detecting Delta (overall) (Fig. 2E). Moreover, the ROC AUCs for the assays ranged between 0.996 and 1.000 (AUC = 0.999, p < 0.001), denoting excellent performance for FinecareTM in detecting Delta (B.1.617.2) (Fig. 2F). In the same way, The ROC AUCs for the assays ranged between 0.901 and 0.986 (AUC = 0.944, p < 0.001), denoting excellent performance for Finecare™ in detecting Alpha variant (Fig. 2G). The ROC AUCs for the assays ranged between 0.957 and 0.991 (AUC = 0.974, p < 0.001), denoting excellent performance for Finecare™ in detecting all SARS-CoV-2 assessed variants (Fig. 2H).

**Fig. 3:**
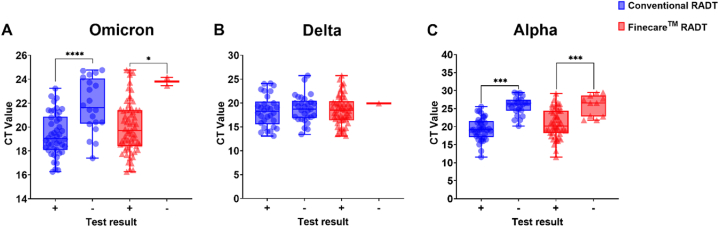
Box blot representing RADT among SARS-CoV-2 variant against Ct value, A) Omicron, B) Delta, and C) Alpha. Significant level × p ≤ 0.05, **p ≤ 0.01, or ***p ≤ 0.001.

Because Ct values are inversely related to viral load, we investigated the RT-qPCR Ct values across SARS-CoV-2 variants using the conventional RADT and Finecare™ RADT (Fig. 3) [28]. Ct values were significantly higher (p ≤ 0.001) among individuals with false-negative conventional RADT compared to true positives across Omicron samples (Fig. 3A). Similarly, Ct values were significantly higher (p ≤ 0.05) among individuals with false-negative Finecare™ RADT compared to true positives across Omicron samples (Fig. 3A). In relation to the Alpha sample, Ct values were significantly higher (p ≤ 0.0001) among individuals with a false-negative conventional RADT compared to true positives across Omicron samples (Fig. 3C). Similar results were obtained from Finecare™ RADT (p ≤ 0.001) (Fig. 3A). On the contrary, Delta samples didn't show any significance using both the conventional RADT and Finecare™ RADT (Fig. 3B).

### 3.4 Rapid antigen detection tests (RADT) performance in the detection of omicron BA.4 and BA.5 lineages

**Fig. 4:**
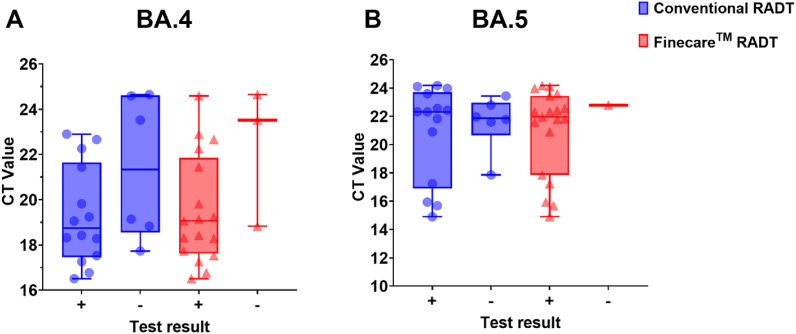
Box blot representing RADT among SARS-CoV-2 variant against Ct value, A) BA.4, and B) BA.5. Significant level × p ≤ 0.05, **p ≤ 0.01, ***p ≤ 0.001.

Furthermore, we evaluated the performance of RADTs in the diagnosis of BA.4 (n = 20) and BA.5 (n = 20) lineages as they harbor unique mutations, including changes referred to as L452R and F486V in the viral spike protein that may weaken their ability to latch onto host cells (Table S2). Ct values showed no significant difference among individuals with false-negative compared to true positives in both conventional RADT test and Finecare™ RADT across BA.4 and BA.5 samples (Fig. 4A and B).

## 4 Discussion

Rapid identification of SARS-CoV-2-infected individuals is essential to limit viral transmission [29]. So far, several RADTs have been developed by various manufacturers from multiple countries, mainly based on either colloidal gold- or immunofluorescence-based assays. Nevertheless, a comparative approach investigating the performance of both types of RADTs in the context of the newly emerged SARS-CoV-2 variants is lacking. In the current study, we evaluated the performance of Finecare™ RADT for the detection of different SARS-CoV-2 variants using the RT-PCR test results as a reference test.

Finecare™ RADT showed an overall better performance compared to conventional RADT (Table 1, Fig. 1) in detecting Omicron, Delta, and Alpha SARS-CoV-2 variants, with an overall sensitivity of 92.6%, and a PPV of 92.6%, in comparison to the conventional RADT (sensitivity: 62.4%, PPV: 62.4%). Nevertheless, the conventional RADT showed higher specificity (specificity: 100%, NPV: 100%) compared to Finecare™ RADT (specificity: 96%, NPV: 96%) (Table 1). According to the WHO, the accepted sensitivity and specificity are ≥80% and ≥97%, respectively, while the European Center for Disease Prevention and Control recommends ≥90% sensitivity and ≥97% specificity [30,31]. This has been a challenging task because almost all of the currently available RADTs were reported to have sensitivity disadvantages. For instance, a recent meta-analysis which evaluated the overall performance of immunofluorescent-based and colloidal gold-based-based RADTs, reported an overall high pooled specificity of ∼99.4%, but poor sensitivity of ∼68.4% [10]. Similarly, a systematic review conducted by Lee et al., revealed an overall sensitivity of 68% [32]. Furthermore, another review summarized the accuracy of multiple RADTs, reporting an average sensitivity of 72.0% in symptomatic individuals [10]. In addition, a study by Freire et al. [33], reported an overall Ag-RDTs sensitivity ranging from 9.8 to 81.1%, and a sensitivity of ∼47.2% for Celler Wondfo SARS-CoV-2 Ag-RADT. It should be noted that such variation in results could be due to several factors, including the type of RADT (fluorescent-based vs. conventional colorimetric), CT values, sample type, type of media used for storage, and sample volume among others.

It is noteworthy to mention that the sensitivity of both RADTs in the current study could be underestimated because nasopharyngeal swab samples were collected in UTM which diluted the sample. Also, for both RADTs, the nasopharyngeal swab samples collected in UTM where further diluted into the RADTs buffer, rather than directly performing the test from the patient nasopharyngeal swab. Besides the fact that samples were stored in −80 °C for over a year. Furthermore, we used a limited sample volume rather than the whole sample. Nonetheless, compared to other RADTs that have been investigated worldwide, the fluorescent based-Finecare™ RADT seems to be top-performing in terms of achieving high sensitivity (92.6%) and high specificity (96%), despite the indicated limitations (Table 1), and thus, Finecare™ could serve as a promising alternative to RT-PCR for SARS-CoV-2 diagnosis. In our previous study [serological], Finecare™ also showed excellent correlation with the surrogate virus-neutralizing test (sVNT, GenScript Biotech, USA) and the VIDAS®3 automated assay (BioMérieux, France), and thus could also serve as a surrogate to assess binding and neutralizing antibody response after infection or vaccination, particularly in none or small laboratory settings [34].

In the current study, we further investigated the sensitivity in relation to the Ct-value. The sensitivity of both Finecare™ RADT decreased dramatically with increasing Ct-value (decreasing viral load) (Table 2, Fig. 3). For Ct-values <20, 20–25, and >25, the conventional RADT showed sensitivities of 75%, 57%, and 6%, respectively, while Finecare™ RADT showed sensitives of 96%, 91%, and 58.8%, respectively (Table 2, Fig. 3). False-negative results were observed across high CT values (low viral load) (Table 2). These findings are in concordance with other studies where a negative correlation of Ct values of RT-PCR and RADTs sensitivity was identified [32]. Currently, there is no definitive Ct-value threshold beyond which antigen tests consistently yield false-negative results. However, a recent study revealed that RADTs are frequently negative in PCR-positive samples with Ct-values above 24–28 [35]. Similarly, another study comprising 468 samples from a German maximum care hospital identified Ct-values ≤22 as the limit for a 100% correlation of PCR and RADT [36]. Based on these findings, early detection using RADTs kits is recommended. Nevertheless, according to the Centers for Disease Control and Prevention (CDC), a higher Ct value (>33) reflects a non-contagious stage [37], which justifies the use of RADTs kits. In a recent study [38], Chao et al. evaluated the performance of a COVID-19 RAT and its correlation with virus infectivity, where cytopathic effects (CPE) in cell culture was used as a reference method for virus infectivity. They demonstrated that only when the Ct values of specimens were below 25, the CPE and RAT results had high degree of consistency.

The emergence of SARS-CoV-2 variants requires an investigation of its potential impact on the performance of diagnostic tests in use, including RADTs. In the current study, we further investigated the performance of the two RADTs for the detection of different SARS-CoV-2 variants and sub-variants. The conventional RADT was able to detect all assessed SARS-CoV-2 variants except for five Delta lineages (AY.106, AY.114, AY.120, AY.34, and AY.5.2). Nevertheless, Finecare™ RADT detected all SARS-CoV-2 variants except for one Delta lineage (AY.34) (Table 3). Both RADTs successfully detected circulating SARS-CoV-2 VOC/I BA.4 and BA.5 (Table 3, Fig. 4). The reason why Finecare™ RADT was able to detect more SARS-CoV-2 variants than the conventional RADT could be due to the fact that Finecare™ RADT is coupled with an FIA meter, which might be associated with a lower limit of detection compared to other conventional RADTs [39]. Few studies investigated RADT performance for the detection of different SARS-CoV-2 variants, but mainly using conventional-colorimetric RADT and not fluorescence [40]. The sensitivity of conventional Wondfo-RADT for the detection of Delta variants was recently reported to be ∼68.5% [40]. This value is comparable but slightly higher than the sensitivity reported in our study for the same type of RADT (Wondfo conventional RADT: Delta; ∼49%). Nevertheless, it should be noted that the exact Delta lineages assessed in this study were unspecified [40].

The main strength of this study lies in the fact that we investigated the performance of RADT against multiple SARS-CoV-2 lineages, including Alpha, Delta, and Omicron variants (Table 3). In addition, we compared the performance of a conventional colorimetric RADT and a new fluorescence-based RADT for the detection of the different lineages.

Despite the strength of this study, there were several limitations. First, our findings were limited to circulating SARS-CoV2 lineages at the time of the investigation. Alpha specimens were not sent for NGS; they were assumed to be Alpha based on the swab collection date. Due to missing clinical manifestations, no distinction was made between symptomatic and non-symptomatic participants, which may influence the sensitivity of this rapid antigen assay. After RT-PCR, samples were frozen in UTM and examined for this study after a period of time that might lead to antigen dilution and degradation, which may have resulted in more false negatives since freeze-thawing may affect sample quality.

## 5 Conclusion

In conclusion, RADTs successfully detected all SARS-CoV-2 variants with high viral loads. Due to the test's high sensitivity and specificity, the immunofluorescence-based Finecare™ RADT is suitable for both clinical and community-based surveillance. Moreover, considering the short turnaround time, user-friendliness, and low costs we believe that in the context of community-based surveillance of symptomatic individuals, these advantages outweigh the lower sensitivity of Wonfo 2019-nCoV-2 Antigen test compared to RT-qPCR.

## Author contribution statement

Gheyath K. Nasrallah: Conceived and designed the experiments; Analyzed and interpreted the data; Contributed reagents, materials, analysis tools or data.

Fatma Ali: Performed the experiments; Analyzed and interpreted the data; Contributed reagents, materials, analysis tools or data.

Salma Younes: Analyzed and interpreted the data; Contributed reagents, materials, analysis tools or data.

Heba A. Al-Khatib; Asmaa A. Al-Thani: Contributed reagents, materials, analysis tools or data.

Hadi M. Yassine: Conceived and designed the experiments; Contributed reagents, materials, analysis tools or data.

## Data availability statement

Data will be made available on request.

## Declaration of competing interest

The authors declare that they have no known competing financial interests or personal relationships that could have appeared to influence the work reported in this paper
