# Supplementary material for: Enhancing the sensitivity of rapid antigen detection test (RADT) of different SARS-CoV-2 variants and lineages using fluorescence-labeled antibodies and a fluorescent meter
Source: Heliyon. 2023 Jun 10;9(6):e17179. doi: 10.1016/j.heliyon.2023.e17179 (PMC10257515; doi:10.1016/j.heliyon.2023.e17179)
Supplement: Multimedia component 1 [file mmc1.docx]

**Supplementary Materials**

**Enhancing the sensitivity of rapid antigen detection test (RADT) of different SARS-CoV-2 variants and lineages using fluorescence-labeled antibodies and a fluorescent meter**

Gheyath K. Nasrallah^1,2,☨^, Fatma Ali^1,2,☨^, Salma Younes^1,2^, Heba A. Al-Khatib^1^, Asmaa A. Al-Thani^1^, Hadi M. Yassine^1,2^*

*^1^Biomedical Research Center, Qatar University, Doha P.O. Box 2713, Qatar.*

*^2^Biomedical Sciences Department, College of Health Sciences, Qatar University, Doha P.O. Box 2713, Qatar.*

^☨^*Equal contribution*

*** Correspondence:** Hadi M. Yassine

Department of Biomedical Science, College of Health Sciences, Qatar University, Doha 2713, Qatar. Tel: +974 4403 6819, Email: [hyassine@qu.edu.qa](mailto:hyassine@qu.edu.qa)

***Table S1.*** *Demographic Data of selected samples.*

| Sample | Omicron  (n=68) | Delta  (n=59) | Alpha  (n=60) | Flu  (n-60) | RSV  (n=60) |
| --- | --- | --- | --- | --- | --- |
| Median age in years (IQR) | 35 (21-53) | 33 (18-45) | 36 (29-45) | 28 (9-38) | 5 (4-5) |
| Gender |  |  |  |  |  |
| Male | 37 (53.6%) | 31 (51.7%) | 45 (75%) | 30 (50%) | 30 (50%) |
| Female | 32 (46.4%) | 29 (48.3%) | 15 (25%) | 30 (50%) | 30 (50%) |
| Region |  |  |  |  |  |
| MENA | 31 (44.9%) | 28 (46.7%) | 25 (41.7%) |  | 51 (85%) |
| Non-MENA | 38 (55.1%) | 32 (53.3%) | 35 (58%) |  | 9 (15%) |
| Not provided |  |  |  | 60 (100%) |  |

MENA: Middle East and North Africa, IQR: Interquartile range.

***Table S2.*** *Demographic Data of Omicron BA.4 and BA.5 lineages*

| Omicron Lineage | BA.4  (n=20) | BA.5  (n=20) |
| --- | --- | --- |
| Median age in years (IQR) | 42 (26-45) | 31 (17-50) |
| Gender |  |  |
| Male | 8 (40%) | 10 (50%) |
| Female | 12 (60%) | 10 (50%) |
| Region |  |  |
| MENA | 9 (45%) | 10 (50%) |
| Non-MENA | 11 (55%) | 10 (50%) |
